# Bilirubin statistically accounts for the association between *Enterobacterales* lineage and Mayo endoscopic score in ulcerative colitis: a cross-sectional associational variable analysis

**DOI:** 10.3389/fmed.2026.1946063

**Published:** 2026-10-14

**Authors:** Zhengyu Ren, Junni Ma, Siyao Wang, Pumeng Fu, Ruixue Wang, Kai Liu, Yingchao Li

**Affiliations:** 1 Department of Gastroenterology, The First Affiliated Hospital of Xi’an Jiaotong University, Xi’an, China; 2 Liver Transplantation Center, West China Hospital, Sichuan University, Chengdu, China

**Keywords:** associational variable analysis, bilirubin, *Enterobacterales* lineage, Mayo endoscopic score, ulcerative colitis

## Abstract

**Background:**

Patients with ulcerative colitis (UC) frequently exhibit gut enrichment of *Enterobacteriaceae* and reduced serum bilirubin levels. However, whether bilirubin statistically accounts for the association between *Enterobacteriaceae* abundance and UC disease activity remains unclear.

**Methods:**

This study enrolled 81 UC patients. The relative abundances of *Enterobacterales* lineage (*Gammaproteobacteria*, *Enterobacterales*, *Enterobacteriaceae*, and *Escherichia-Shigella*) were determined by 16S rRNA sequencing, and disease activity was assessed using the Mayo endoscopic score (MES). Total bilirubin (TBIL) and indirect bilirubin (IBIL) were examined as potential associative variables. Associational variable analyses were performed using bootstrap methods with 5,000 resamples, and sensitivity analyses were conducted after adjusting for covariates.

**Results:**

The abundances of *Enterobacterales* lineage were positively correlated with MES (*p* = 0.34–0.40) and negatively correlated with TBIL and IBIL (*p* = −0.31 to −0.48). Associational variable analyses showed that bilirubin significantly and partially accounted for these positive associations. In covariate-adjusted models, the associational proportions ranged from 40.8% to 42.3% for TBIL and from 37.2% to 40.0% for IBIL.

**Conclusion:**

Bilirubin statistically accounts for approximately 37.2%–42.3% of the association between *Enterobacterales* lineage abundance and UC disease activity. Owing to the cross-sectional design, these findings reflect statistical covariation rather than causal effects, and prospective studies are needed to establish temporal direction.

## 1 Introduction

Ulcerative colitis (UC) is a chronic inflammatory disease of the colorectal mucosa, characterized by a relapsing–remitting course that substantially impairs patients' quality of life. With its global prevalence continuing to rise in newly industrialized regions, UC imposes a growing health burden worldwide, underscoring the need for further research into factors associated with its disease activity (1, 2).

Gut microbiota dysbiosis has emerged as a key contributor to UC pathogenesis. Patients with UC exhibit marked enrichment of the phylum Proteobacteria and particularly the family *Enterobacteriaceae* (3–5). This enrichment is one of the most consistently observed microbial alterations in active UC. Cross-sectional studies have associated *Enterobacteriaceae* abundance with disease activity, and pathogenic strains are more abundant in the inflamed UC gut (4, 6). Patients with higher baseline *Enterobacteriaceae* levels go on to face a greater risk of biologic therapy failure within 5 years (7). In animal models, expanding *Enterobacteriaceae* worsens colitis, while eliminating them reduces inflammation (8, 9).

Bilirubin, the end product of heme breakdown, was long viewed as metabolic waste, but at physiological concentrations it exerts antioxidant and anti-inflammatory effects (10, 11). In UC, serum bilirubin is lower than in healthy controls, correlates inversely with disease activity, and rises with effective treatment (12). Current data on the relationship between bilirubin and *Enterobacteriaceae* come mainly from neonatal studies and are conflicting; some report a positive correlation between *Enterobacter* abundance and bilirubin (13, 14), while others find an inverse association (15, 16). Thus, whether bilirubin statistically accounts for the *Enterobacteriaceae* and disease activity association in UC has not been examined.

Therefore, we performed a cross-sectional associational variable analysis using the *Enterobacterales* lineage (from class *Gammaproteobacteria* to genus *Escherichia-Shigella*) as the primary variable, Mayo endoscopic score (MES) as the dependent variable, and bilirubin as associative variables. Additional taxa within *Gammaproteobacteria* were also examined descriptively to provide a broader compositional context, aiming to investigate whether bilirubin statistically accounts for the *Enterobacterales*–disease activity association.

## 2 Materials and methods

### 2.1 Patients and clinical data collection

This study enrolled patients with UC hospitalized in the Department of Gastroenterology at the First Affiliated Hospital of Xi'an Jiaotong University from May 2022 to June 2024. UC diagnosis was confirmed by gastroenterologists based on endoscopic findings, with histopathological confirmation when available. Eligible patients were aged 20–70 years and provided written informed consent. Exclusion criteria were: (1) use of antibiotics, corticosteroids, or probiotics within three months prior to enrollment; (2) refusal to undergo essential diagnostic procedures; (3) presence of other acute or chronic cardiovascular, gastrointestinal, or immune-mediated diseases; or (4) abnormal liver function tests, defined as elevated serum levels of alanine aminotransferase, aspartate aminotransferase, alkaline phosphatase, or gamma-glutamyl transferase above the upper limit of normal. Demographic and clinical characteristics, including sex, age, body mass index (BMI), total bilirubin (TBIL), indirect bilirubin (IBIL), high-sensitivity C-reactive protein (hs-CRP), erythrocyte sedimentation rate (ESR), disease duration, Montreal classification and MES, were extracted from electronic medical records and supplemented by patient interviews. Therapy history was defined as drug exposure within 3 months before enrollment.

### 2.2 Fecal and blood sample collection and processing

Fasting venous blood (3 mL) was collected on the morning after admission. Blood for serum biochemistry (bilirubin, hs-CRP) was drawn into plain red-top tubes (no anticoagulant), while blood for ESR was collected into black-top tubes containing 3.2% sodium citrate. All tubes were transported to the clinical laboratory within 2 h. Biochemical measurements were performed within 6 h whenever possible; if not, samples were stored at 4 °C and analyzed within 24 h.

Fecal samples (approximately 5 g) were collected by patients or family members using sterile spoons, immediately after written informed consent was obtained. Samples were placed into sterile cryotubes labeled with unique identification codes, with care taken to avoid contact with urine, toilet water, or other potential contaminants. Routine sterile cryotubes without DNA stabilizers were used. Within 2 h of collection, samples were placed on ice, transported under cold-chain conditions to the hospital biobank, and directly snap-frozen at −80 °C. Repeated freeze-thaw cycles were strictly avoided throughout to minimize changes in microbial composition prior to analysis.

### 2.3 16S rRNA gene sequencing and microbiota analysis

Fecal microbial DNA was extracted from approximately 0.2 g of each sample using the HiPure Stool DNA Kit (Magen, Guangzhou, China). Samples were lysed chemically in 1.2 mL of lysis buffer at 70 °C for 10 min, followed by centrifugation, proteinase K digestion, and spin-column purification. No mechanical lysis or bead beating was performed. DNA concentration and purity were assessed using a NanoDrop 2000 spectrophotometer, and DNA integrity was examined by agarose gel electrophoresis. The V3–V4 region of the bacterial 16S rRNA gene was amplified using barcoded primers 341F (5′-CCTACGGGNGGCWGCAG-3′) and 806R (5′-GGACTACHVGGGTATCTAAT-3′). PCR was performed in 50 μL mixtures containing 5 μL of 10× KOD buffer, 5 μL of 2 mM dNTPs, 3 μL of 25 mM MgSO4, 1.5 μL of each primer (10 μM), 1 μL of KOD DNA polymerase, and 100 ng of template DNA. The cycling protocol consisted of initial denaturation at 94 °C for 2 min; 30 cycles of 98 °C for 10 s, 65 °C for 30 s, and 68 °C for 30 s; and a final extension at 68 °C for 5 min. Purified amplicons were quantified, pooled at equimolar concentrations, and sequenced using 2 × 250-bp paired-end sequencing on an Illumina MiSeq platform. The PCR amplification was performed on an ETC811 thermal cycler (Dongsheng Xingye, China).

Raw reads were quality-filtered using FASTP v0.20.0, merged using FLASH v1.2.11 with a minimum overlap of 10 bp, and further filtered according to sequence length and quality. OTUs were clustered at 97% similarity using UPARSE in USEARCH v11.0.667. Chimeric sequences were removed using UCHIME v4.2.40 against the SILVA database, and taxonomy was assigned using the naïve Bayesian classifier with a confidence threshold of 0.7 and SILVA v138. Negative controls were used to screen for potential contaminants, and mitochondrial and chloroplast sequences were removed. The OTU table was rarefied to the minimum sequencing depth before downstream analyses.

### 2.4 Statistical analysis

Normally distributed variables are presented as mean ± SD, non-normally distributed variables as median (IQR), and categorical variables as numbers (percentages). Since relative abundance data of bacterial taxa are highly skewed, all abundance values were transformed using log_10_(1 + relative abundance percentage) to stabilize variance, reduce the influence of extreme values, preserve zero values, and facilitate subsequent association analyses. Original zero values therefore mapped to 0 on the transformed scale. No imputation was performed. This transformation was applied for univariate descriptive and associational purposes rather than for multivariate compositional analyses. Considering that relative abundance data derived from 16S rRNA sequencing may exhibit interdependencies among taxa within the same group, we additionally selected other *Gammaproteobacteria*-associated taxa to describe the compositional context. These taxa were required to meet the following prespecified criteria: non-zero detection frequency >95% and mean relative abundance >0.1%. Normality of continuous variables was assessed using the Shapiro–Wilk test; Spearman's rank correlation was used to evaluate associations among gut microbiota abundances, bilirubin, and MES. The Benjamini–Hochberg false discovery rate (FDR) correction was applied to correlation analyses involving multiple variables; an FDR-adjusted *q* < 0.05 was considered significant.

Post-hoc statistical power was evaluated using G*Power (version 3.1.9.7) for multiple linear regression (*F* tests) (17). The analysis was specified with a sample size of 81, nine predictors (including associational variable), a two-sided significance level of *α* = 0.05, and an effect size (*f*^2^) calculated from the observed R² of each MES outcome model [*f*^2^ = *R*^2^/(1 − *R*^2^)]. Statistical power was then computed for each model.

To interpret the cross-sectional findings without causal overstatement, we adopted the associational variable analysis framework proposed by Weems (18). The schematic diagram of the analytical model is shown in Figure 1. We performed a regression-based associational decomposition analysis using SPSS PROCESS (Model 4). Sensitivity analyses were performed to adjust for other covariates, including age, sex, BMI, smoking history, disease duration, biologic therapy history and Montreal classification. In a separate exploratory model, we further adjusted for inflammatory markers (hs-CRP and ESR). The robustness of the sensitivity analyses was predefined as a change in the associative proportion of less than 10% after adjustment. Collinearity between each bacterial taxon and bilirubin, as well as between each bacterial taxon and MES, was assessed using variance inflation factor (VIF), tolerance, and condition index. Two-sided tests were used, and *P* < 0.05 was considered statistically significant. Statistical analyses were performed using SPSS 26.0 (IBM, USA), and figures were generated with R 4.6.0.

**Figure 1:**
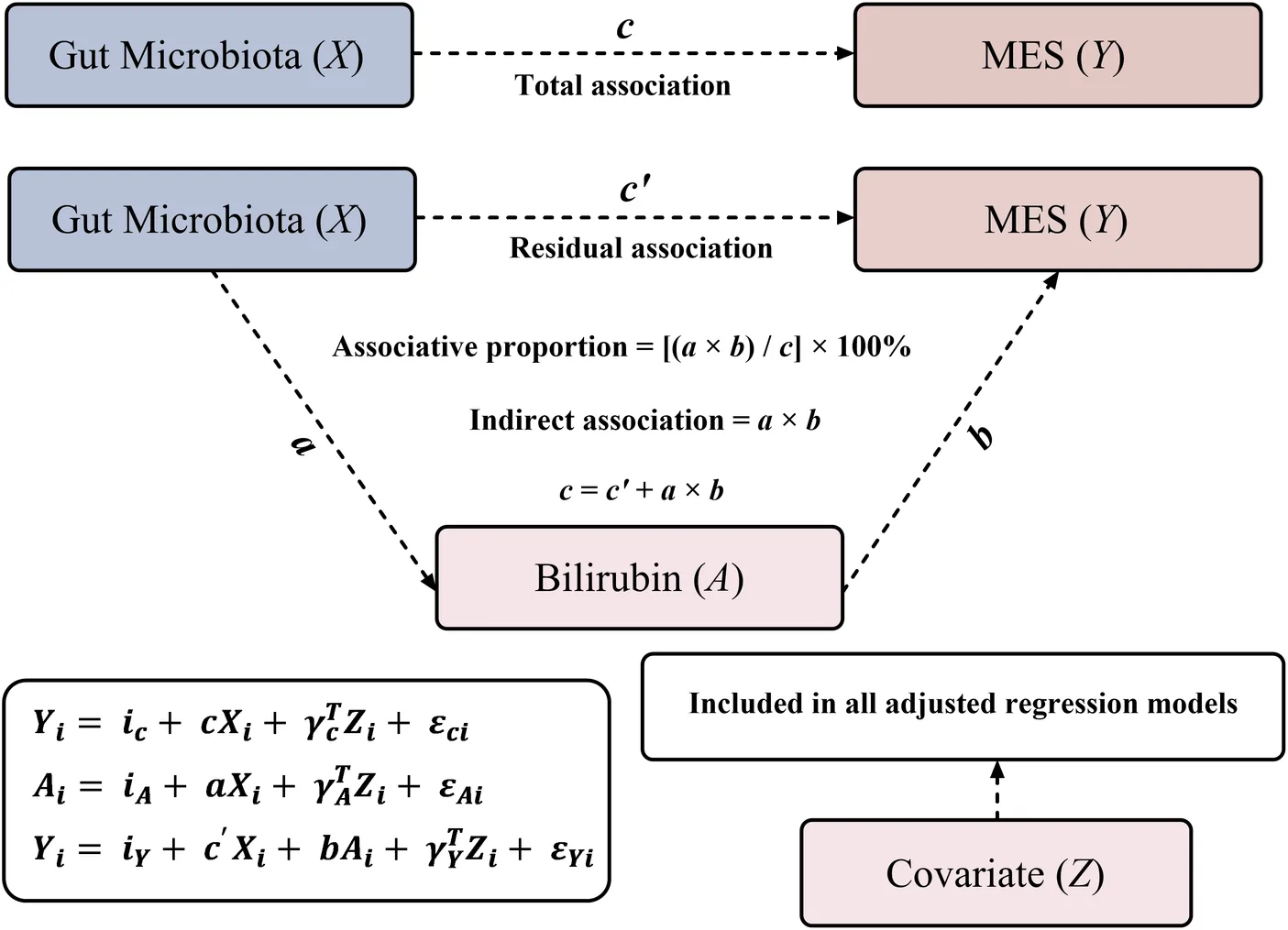
Regression-based associational decomposition framework for the relationship between gut microbiota, bilirubin, and Mayo endoscopic score (MES). Gut microbiota was represented by *X*, bilirubin was represented by *A*, and MES was represented by *Y*. The coefficient *c* denotes the total association between gut microbiota and MES after adjustment for the prespecified covariates *Z*. The coefficient *c’* denotes the residual association between gut microbiota and MES after accounting for bilirubin and the covariates. The coefficient *a* denotes the adjusted regression association between gut microbiota and bilirubin. The coefficient *b* denotes the adjusted regression association between bilirubin and MES after accounting for gut microbiota and the covariates. The associational proportion was calculated as [(*a* × *b*)/*c*] × 100%, and the indirect association was calculated as *a* × *b*, as shown in the figure. For linear regression models fitted to the same analytic sample with the same covariates, the coefficients satisfy the algebraic relationship *c* = *c’* + *a* × *b*. The three regression equations shown in the figure define the total association model, the gut microbiota and bilirubin association model, and the mutually adjusted MES model, respectively. In these equations, *i* denotes the individual participant, $X_{i}$ denotes the gut microbiota measurement for participant *i*, $A_{i}$ denotes the bilirubin measurement for participant *i*, $Y_{i}$ denotes the MES value for participant *i*, and $Z_{i}$ denotes the vector of covariates for participant *i*. The terms $i_{c}$, $i_{A}$, and$\;i_{Y}\;$denote the intercepts of the total association model, the gut microbiota and bilirubin association model, and the mutually adjusted MES model, respectively. The terms $\gamma_{c}$, $\gamma_{A}$, and $\gamma_{Y}$ denote the vectors of regression coefficients for the covariates in the corresponding models. The superscript *T* denotes matrix transposition. The terms $\gamma_{c}^{T}Z_{i}$, $\gamma_{A}^{T}Z_{i}$, and $\gamma_{Y}^{T}Z_{i}$ represent the combined contribution of the covariates in the corresponding regression models. The terms $\varepsilon_{ci}$, $\varepsilon_{Ai}$, and $\varepsilon_{Yi}$ denote the residual terms for participant *i* in the corresponding models. This framework describes statistical associations and does not establish causal effects or causal mediation.

## 3 Results

### 3.1 Baseline characteristics of the study population

A total of 81 UC patients were included after excluding 40 of 121 initially assessed patients due to inadequate fecal samples or missing concurrent laboratory data. The median age was 48.0 years (IQR: 35.0–61.0), with 59.3% being male, and the median disease duration was 60.0 months (IQR: 24.0–120.0). The mean BMI was 21.9 ± 3.5 kg/m^2^. Smoking history was reported in 13 patients (16.0%). According to the Montreal classification, 29 patients (35.8%) had E1, 19 (23.5%) had E2, and 33 (40.7%) had E3. The distribution of MES scores was 16.0% for score 1, 58.0% for score 2, and 26.0% for score 3. Nearly all patients (97.5%) had mesalazine exposure; biologic therapy history included infliximab (44.4%), vedolizumab (11.2%), and no biologic agent (44.4%). Serum TBIL (median: 9.5 μmol/L; IQR: 6.8–13.5), IBIL (median: 5.4 μmol/L; IQR: 3.7–8.5), hs-CRP (median: 3.1 mg/L; IQR: 0.8–8.0), and ESR (median: 15.0 mm/h; IQR: 7.0–23.5) were all significantly non-normally distributed (Shapiro–Wilk *P* < 0.001 for all). In contrast, BMI was normally distributed (Shapiro–Wilk *P* = 0.160) (Table 1).

**Table 1:** Baseline clinical characteristics of the study population (*n* = 81).

Variable	*N* (%)	Mean ± SD	Median (IQR)	Shapiro–wilk *P*
Demographic characteristics				
Age (y)			48.0 (35.0–61.0)	0.034
Sex (male, %)	48 (59.3%)			
BMI (kg/m^2^)		21.9 ± 3.5		0.160
Smoking (yes, %)	13 (16.0%)			
Disease duration (m)			60 (24–120)	<0.001
Bilirubin levels				
TBIL (μmol/L)			9.5 (6.8–13.5)	<0.001
IBIL (μmol/L)			5.4 (3.7–8.5)	<0.001
Inflammatory level				
hs-CRP (mg/L)			3.1 (0.8, 8.0)	<0.001
ESR (mm/h)			15.0 (7.0, 23.5)	<0.001
Montreal classification				
E1	29 (35.8%)			
E2	19 (23.5%)			
E3	33 (40.7%)			
Disease activity				
MES (1, %)	13 (16.0%)			
MES (2, %)	47 (58.0%)			
MES (3, %)	21 (26.0%)			
Mesalazine history				
Yes	79 (97.5%)			
No	2 (2.5%)			
Biologic therapy history				
No biologic agent	36 (44.4%)			
Infliximab	36 (44.4%)			
Vedolizumab	9 (11.2%)			

TBIL, total bilirubin; IBIL, indirect bilirubin; hs-CRP, high-sensitivity C-reactive protein; ESR, erythrocyte sedimentation rate. MES, Mayo endoscopic score.

Data are presented as mean ± SD, median (IQR), or *N* (%). The Shapiro–Wilk test was used to assess normality; All variables had complete data (*n* = 81). *P* < 0.05 indicates deviation from normal distribution.

### 3.2 Compositional context of *Gammaproteobacteria*-associated taxa

Across the 81 samples, the number of retained effective reads, effective-read ratio, and OTU counts per sample are reported (Supplementary Tables S1, S2). Among the four prespecified taxa, *Gammaproteobacteria*, *Enterobacterales*, and *Enterobacteriaceae* were the most abundant, with raw relative abundances of 15.44%, 12.81%, and 12.43%, respectively, and were universally detected across all samples. *Escherichia–Shigella* also showed a 100% detection rate, with a raw relative abundance of 8.14%. After log₁₀ transformation, their mean relative abundances were 0.98 ± 0.42, 0.85 ± 0.51, 0.85 ± 0.48, and 0.71 ± 0.45, respectively. These four taxa were retained as the primary analysis variables. To minimize potential biases arising from the compositional nature of 16S rRNA sequencing data and provide a broader compositional context, we included five additional *Gammaproteobacteria*-associated taxa that met the prevalence (>95%) and mean relative abundance (>0.1%) filters: *Burkholderiales* (Order), *Pasteurellales* (Order), *Pseudomonadales* (Order), *Morganellaceae* (Family), and *Citrobacter* (Genus) (Supplementary Table S3). Among the contextual taxa, *Citrobacter* was detected in 97.5% of samples, with a raw relative abundance of 0.22%, while the remaining taxa showed lower abundances and detection rates (Table 2). On the transformed scale, *Gammaproteobacteria* was the only taxon with a normal distribution (Shapiro–Wilk *P* = 0.156), while the other eight taxa deviated significantly from normality (*P* < 0.05).

**Table 2:** Gut microbiota descriptive statistics.

Gut microbiota	Non-zero samples	Raw relative abundance (%)	Transformed relative abundance, mean ± SD	Shapiro–wilk *P*
Prespecified *Enterobacterales*-lineage taxa				
*Gammaproteobacteria* (Class)	81	15.435	0.98 ± 0.42	0.156
*Enterobacterales* (Order)	81	12.805	0.85 ± 0.51	0.034
*Enterobacteriaceae* (Family)	81	12.429	0.85 ± 0.48	0.007
*Escherichia-Shigella* (Genus)	81	8.141	0.71 ± 0.45	0.002
Contextual taxa				
*Burkholderiales* (Order)	81	1.436	0.31 ± 0.28	<0.001
*Pasteurellales* (Order)	79	0.581	0.14 ± 0.20	<0.001
*Pseudomonadales* (Order)	77	0.281	0.08 ± 0.15	<0.001
*Morganellaceae* (Family)	78	0.356	0.07 ± 0.17	<0.001
*Citrobacter* (Genus)	79	0.224	0.06 ± 0.13	<0.001

Microbial abundances were transformed as log_10_(1 + relative abundance %). The “Raw relative abundance (%)” column shows the original percentage before transformation. Transformed values are dimensionless. Shapiro–Wilk *P* < 0.05 indicates non-normal distribution.

### 3.3 Associations of prespecified and contextual taxa with MES and bilirubin measures

We evaluated associations between gut microbiota (including the four prespecified *Enterobacterales* lineage taxa and the five contextual taxa), MES, and serum bilirubin using Spearman correlation analysis after transformation of taxon abundances. Initially, we examined the relationship between MES and bilirubin levels. MES was inversely correlated with both TBIL (*ρ* = −0.55, *P* < 0.001) and IBIL (*ρ* = −0.48, *P* < 0.001) (Figure 2). Subsequently, after Benjamini–Hochberg correction across 27 tests, the *Enterobacterales* lineage (from class *Gammaproteobacteria* to genus *Escherichia–Shigella*) showed the most consistent associations, with positive correlations with MES (*ρ* = 0.34–0.40) and negative correlations with TBIL (*ρ* = −0.35 to −0.46) and IBIL (*ρ* = −0.31 to −0.48). Among the contextual taxa, only *Citrobacter* showed a significant association with MES (*ρ* = 0.29, *q* < 0.05), whereas the remaining taxa showed weak or inconsistent relationships with the clinical measures (Figure 3).

**Figure 2:**
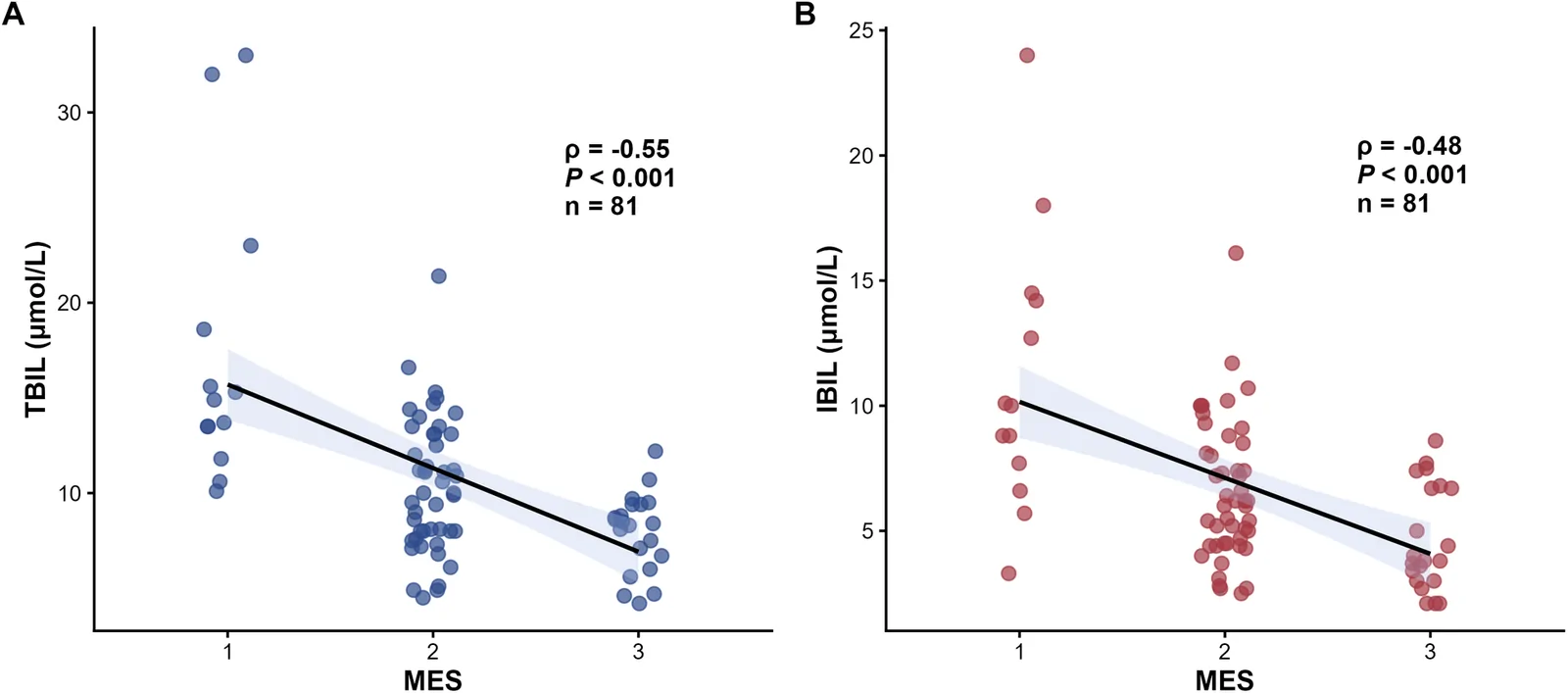
Scatter plots of total bilirubin (TBIL) and indirect bilirubin (IBIL) across Mayo endoscopic score (MES) groups. **(A)** Correlation between TBIL and MES. **(B)** Correlation between IBIL and MES. Each dot represents an individual patient (*n* = 81), with colors indicating MES category (orange: MES 1; blue: MES 2; red: MES 3). Solid blue lines represent linear regression fits with 95% confidence intervals (shaded areas). Spearman correlation coefficients (*p*), *P*-values, and sample sizes are displayed in the upper right corner of each panel.

**Figure 3:**
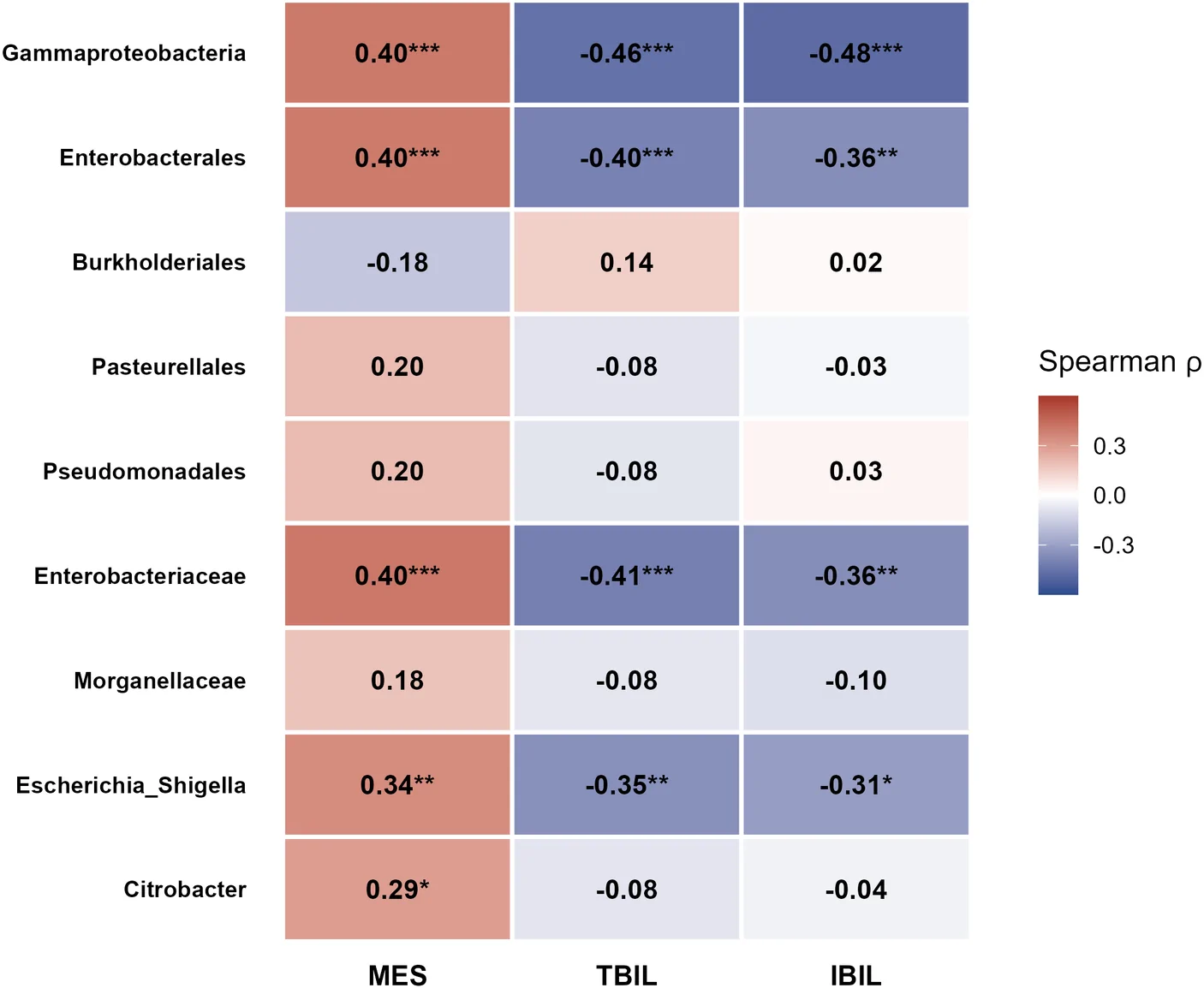
Spearman correlation heatmap between the nine eligible taxa and Mayo endoscopic score (MES), total bilirubin (TBIL), and indirect bilirubin (IBIL). Cell colors and values indicate Spearman correlation coefficients (rho), with red indicating positive correlations and blue indicating negative correlations. Benjamini–Hochberg false-discovery-rate adjustment was applied across all 27 taxon–clinical variable correlation tests. *q < 0.05; **q < 0.01; ***q < 0.001.

### 3.4 Associational variable analysis

We next performed associational decomposition analyses to quantify the statistical role of bilirubin in the associations between the four prespecified taxa and MES. In unadjusted associational models, the positive associations between the *Enterobacterales* lineage and MES, and the inverse associations of TBIL and IBIL with MES, were further confirmed by regression coefficients. Indirect associative effects (*a* × *b*) ranged from 0.16 to 0.25, corresponding to 31.4%–37.9% of the total associations. After adjustment for age, sex, BMI, smoking history, disease duration, biologic therapy history, and Montreal classification, the results remained materially unchanged. Adjusted indirect effects ranged from 0.18 to 0.27, with bootstrap 95% CIs excluding zero, and accounted for 37.2%–42.3% of the total associations (Table 3). Collinearity diagnostics for the eight adjusted MES outcome models showed maximum VIFs of 1.200–1.232, minimum tolerance values of 0.812–0.833, and maximum condition indices of 29.125–30.163. The corresponding variance proportions were primarily attributable to the intercept and BMI, with no dimension showing high variance proportions for two or more substantive predictors (Table 4).

**Table 3:** Associational analysis of gut microbiota in the relationship between serum bilirubin and Mayo endoscopic score, with covariate-adjusted sensitivity analyses.

Variable	*a*	*b*	*c*′	*a* × *b*	*c*	Proportion	
*Gammaproteobacteria* (Class level)							
TBIL	Unadjusted	−4.09 [−6.75, −1.42]	−0.06 [−0.08, −0.04]	0.42 [0.12, 0.72]	0.24 [0.13, 0.40]	0.66	36.4%
	Adjusted	−4.18 [−6.89, −1.47]	−0.07 [−0.09, −0.04]	0.38 [0.07, 0.69]	0.27 [0.12, 0.46]	0.65	41.5%
IBIL	Unadjusted	−3.56 [−5.50, −1.61]	−0.07 [−0.10, −0.04]	0.41 [0.09, 0.73]	0.25 [0.11, 0.44]	0.66	37.9%
	Adjusted	−3.52 [−5.55, −1.48]	−0.08 [−0.11, −0.04]	0.39 [0.06, 0.72]	0.26 [0.11, 0.47]	0.65	40.0%
*Enterobacterales* (Order level)							
TBIL	Unadjusted	−3.02 [−5.29, −0.76]	−0.06 [−0.08, −0.04]	0.35 [0.10, 0.60]	0.19 [0.09, 0.31]	0.54	35.2%
	Adjusted	−3.13 [−5.47, −0.79]	−0.07 [−0.09, −0.04]	0.30 [0.04, 0.56]	0.21 [0.08, 0.35]	0.51	41.2%
IBIL	Unadjusted	−2.45 [−4.13, −0.77]	−0.07 [−0.11, −0.04]	0.36 [0.10, 0.62]	0.18 [0.07, 0.32]	0.54	33.3%
	Adjusted	−2.42 [−4.20, −0.64]	−0.08 [−0.11, −0.04]	0.32 [0.05, 0.60]	0.19 [0.06, 0.34]	0.51	37.2%
*Enterobacteriaceae* (Family level)							
TBIL	Unadjusted	−3.07 [−5.32, −0.81]	−0.06 [−0.08, −0.04]	0.35 [0.10, 0.60]	0.19 [0.09, 0.32]	0.54	35.2%
	Adjusted	−3.21 [−5.54, −0.87]	−0.07 [−0.09, −0.04]	0.30 [0.04, 0.56]	0.22 [0.09, 0.35]	0.52	42.3%
IBIL	Unadjusted	−2.48 [−4.15, −0.81]	−0.07 [−0.11, −0.04]	0.36 [0.10, 0.62]	0.18 [0.07, 0.33]	0.54	33.3%
	Adjusted	−2.48 [−4.25, −0.70]	−0.08 [−0.11, −0.04]	0.32 [0.05, 0.59]	0.19 [0.06, 0.35]	0.51	37.2%
*Escherichia-Shigella* (Genus level)							
TBIL	Unadjusted	−2.57 [−5.04, −0.09]	−0.06 [−0.09, −0.04]	0.35 [0.08, 0.61]	0.16 [0.04, 0.31]	0.51	31.4%
	Adjusted	−2.87 [−5.39, −0.35]	−0.07 [−0.09, −0.04]	0.29 [0.01, 0.56]	0.20 [0.05, 0.34]	0.49	40.8%
IBIL	Unadjusted	−2.23 [−4.06, −0.39]	−0.08 [−0.11, −0.04]	0.34 [0.06, 0.62]	0.17 [0.05, 0.32]	0.51	33.3%
	Adjusted	−2.25 [−4.17, −0.34]	−0.08 [−0.12, −0.05]	0.30 [0.02, 0.59]	0.18 [0.04, 0.35]	0.48	37.5%

TBIL, total bilirubin; IBIL, indirect bilirubin.

The coefficient *a* denotes the adjusted regression association between gut microbiota and bilirubin. The coefficient *b* denotes the adjusted regression association between bilirubin and Mayo endoscopic score (MES) after accounting for gut microbiota and the covariates. The coefficient *c* denotes the total association between gut microbiota and MES. The coefficient *c*′ denotes the residual association between gut microbiota and MES after accounting for bilirubin and the covariates. The indirect association was calculated as *a* × *b.* The 95% CI indicates the uncertainty surrounding the estimated *a* × *b* value and was calculated using 5,000 bootstrap resamples. The associational proportion was calculated as [(*a* × *b*)/*c*] × 100%. The proportional estimate may be numerically unstable when *c* is close to zero or negative and should therefore be interpreted cautiously.

Adjusted models included age, sex, BMI, smoking history, disease duration, biologic therapy history, and Montreal classification.

**Table 4:** Collinearity diagnostics for the MES outcome models.

Variable	Max VIF	Min tolerance	Max CI	High variance proportions at max CI
*Gammaproteobacteria*				
TBIL	1.232	0.812	29.805	Constant 0.94; BMI 0.75
IBIL	1.224	0.817	30.163	Constant 0.95; BMI 0.74
*Enterobacterales*				
TBIL	1.221	0.819	29.390	Constant 0.94; BMI 0.79
IBIL	1.200	0.833	29.610	Constant 0.95; BMI 0.79
*Enterobacteriaceae*				
TBIL	1.223	0.818	29.346	Constant 0.94; BMI 0.79
IBIL	1.200	0.833	29.562	Constant 0.95; BMI 0.79
*Escherichia-Shigella*				
TBIL	1.226	0.816	29.125	Constant 0.94; BMI 0.81
IBIL	1.200	0.833	29.266	Constant 0.95; BMI 0.81

VIF, variance inflation factor; CI, condition index; Max, maximum; Min, minimum.

Values represent the maximum VIF, minimum tolerance, and maximum condition index observed in each covariate-adjusted Mayo endoscopic score (MES) outcome model.

To evaluate the risk of Type II error (*β*), we performed post-hoc power analyses based on the actual observed *R*^2^ values of the MES outcome models, which ranged from 0.362 to 0.428. The corresponding effect sizes (*f*^2^) ranged from 0.568 to 0.749. With a sample size of 81, nine predictors, and *α* = 0.05 (two-tailed), the statistical power ranged from 99.6% to 99.9% (Supplementary Table S5), with corresponding Type II error rates ranging from 0.1% to 0.4%, indicating that the risk of false-negative findings was negligible.

In a separate sensitivity analysis, we further adjusted for inflammatory markers (hs-CRP and ESR). Under this additional adjustment, the bootstrap 95% CIs for all indirect effects crossed zero, indicating that the statistical explanatory role of bilirubin was attenuated to non-significance (Supplementary Table S4).

## 4 Discussion

Gut microbiota dysbiosis, particularly *Enterobacteriaceae* enrichment, is a hallmark of IBD (19–21). We found that *Enterobacterales* lineage (*Gammaproteobacteria*, *Enterobacterales*, *Enterobacteriaceae*, and *Escherichia-Shigella*) was positively correlated with MES, consistent with prior reports linking *Enterobacteriaceae* abundance to UC severity (22, 23). Overgrowth of *Enterobacteriaceae* contributes to UC pathogenesis through LPS-induced TLR4/NF-κB activation (24, 25), and epithelial invasion and intracellular persistence by adherent-invasive *E. coli* (26). In contrast, bilirubin exerts anti-inflammatory effects at physiological concentrations (10), and its serum levels correlate inversely with disease activity in UC (12, 27). Thus, bilirubin and *Enterobacteriaceae* appear to play opposing roles in UC inflammation.

Bilirubin also showed inverse correlations with both *Enterobacteriaceae* and MES. Our study found a negative correlation between *Enterobacterales* lineage taxa and bilirubin. This finding contrasts with previous *in vitro* evidence linking bilirubin to protection of *E. coli* from oxidative stress (10). One possible explanation is the absence of inflammatory consumption *in vitro* vs. its presence *in vivo*. In the UC gut, *Enterobacteriaceae* expansion triggers LPS release and cytokine production (TNF-α, IL-6) (2, 28), while bilirubin is consumed neutralizing reactive oxygen species, reducing its serum levels. The same inflammatory environment also favors *Enterobacteriaceae* outgrowth through increased nitrate availability (29, 30). Thus, although bilirubin may exert direct protective effects on Gram-negative bacteria *in vitro*, this effect is likely overwhelmed by inflammation-driven consumption in the inflamed UC gut, resulting in a net negative correlation. In support of this interpretation, we found that *Enterobacteriaceae* abundance correlated positively with hs-CRP and ESR, whereas bilirubin correlated negatively with these markers (Supplementary Figure S1). In addition, neonatal studies on bilirubin and gut microbiota also have yielded conflicting results. Some report a positive correlation between *Enterobacter* abundance and TBIL (13, 14), while others find decreased abundance of *Gammaproteobacteria*, *Enterobacteriaceae*, and *Escherichia-Shigella* when bilirubin is elevated (15, 16). The degree of bilirubin elevation may explain this discrepancy. Studies showing a negative correlation predominantly involved infants with mild hyperbilirubinemia (e.g., breast milk jaundice), whereas those reporting a positive correlation mainly enrolled neonates with severe pathological jaundice (e.g., hemolytic disease). Bilirubin exerts anti-inflammatory effects only at physiological concentrations, inhibiting NF-κB signaling and NLRP3 inflammasome activation (31, 32); at supraphysiological levels, it becomes cytotoxic, induces pro-inflammatory cytokine release, and may paradoxically promote *Enterobacteriaceae* growth (33). Furthermore, *Enterobacteriaceae* are major producers of β-glucuronidase, an enzyme directly involved in bilirubin metabolism (34, 35). However, direct evidence linking gut microbial β-glucuronidase activity to circulating bilirubin levels in humans remains limited.

The inverse correlation between bilirubin and MES in patients with UC has been reported in several previous studies. Bilirubin has anti-inflammatory properties at physiological concentrations (10). In UC, serum bilirubin correlates inversely with disease activity, and rises with effective treatment (12, 27). Derived ratios such as the C-reactive protein-to-bilirubin ratio and neutrophil-to-bilirubin ratio also correlate with endoscopic activity (36, 37). Exogenous bilirubin alleviates DSS-induced colitis through scavenging reactive oxygen species, inhibiting NLRP3 inflammasome activation, and stabilizing mitochondrial membrane potential (10, 32). Our associational analysis provides the first quantitative estimate of bilirubin's role in the relationship between the *Enterobacterales* lineage and UC disease activity. Approximately 37%–42% of the association between the *Enterobacterales* lineage and MES statistically co varied with bilirubin levels. Notably, adjustment for biologic therapy history, Montreal classification, and other clinical covariates resulted in a slight enhancement of TBIL-related indirect effects, from 31.4% to 36.4% in unadjusted models to 40.8%–42.3% after adjustment, suggesting negative confounding by these covariates. Both biologic therapy history and more extensive disease (e.g., Montreal E3) are associated with greater disease severity and may influence gut microbiota composition, these factors may have partially masked the associative contribution of TBIL in the unadjusted analyses. Biologic therapies have been shown to modulate the gut microbiota in patients with IBD, with infliximab and vedolizumab treatment associated with decreased *Enterobacterales* abundance (38, 39). Given that biologic therapies exert their anti-inflammatory effects by reducing systemic inflammation, one would expect that adjusting for inflammatory markers would attenuate the indirect effects of bilirubin. Consistent with this expectation, when additional adjustment was made for inflammatory markers (hs-CRP and ESR), the bootstrap 95% CIs for all indirect effects crossed zero (Supplementary Table S4), indicating that the statistical explanatory role of bilirubin was attenuated to non-significance. This is consistent with the well-established anti-inflammatory properties of bilirubin (31). This attenuation could reflect that bilirubin's statistical covariation is embedded within systemic inflammatory pathways. Alternatively, adding two further covariates may alter model properties and reduce statistical power, which might also contribute to loss of significance. Thus, bilirubin does not appear to act independently of inflammation in the association between the *Enterobacterales* lineage and disease activity. This inflammatory context may also help explain the similar associative proportions observed between TBIL and IBIL. Although IBIL is recognized as the primary antioxidant and anti-inflammatory component within total bilirubin (32), theoretically, it would be expected to account for a larger associative proportion than TBIL. However, our results did not support this expectation. A possible explanation is that the generally low serum bilirubin levels in our UC cohort, together with the inherent collinearity between TBIL and its indirect fraction, may have reduced the detectable contrast between the two fractions. Whether the distinction between TBIL and IBIL becomes more apparent in populations with higher or more variable bilirubin levels warrants further investigation.

This study has several limitations. First, the cross-sectional design precludes causal inference and bidirectional interactions cannot be ruled out. Although previous studies have established that *Enterobacterales* enrichment is a characteristic feature of UC, the lack of a healthy control group limits assessment of UC-specific microbial patterns and generalizability. Second, the use of 16S rRNA gene sequencing inherently restricts taxonomic resolution to higher ranks (class to genus levels), precluding strain-level identification of specific pathogenic bacteria. Of note, DNA extraction used only heat-assisted chemical lysis without bead-beating, potentially underestimating Gram-positive bacteria and indirectly altering compositional profiles, though bias toward target Gram-negative *Enterobacterales*-related taxa is expected to be modest. Third, *post-hoc* power analysis indicated adequate statistical power to detect the observed effects, suggesting a low risk of Type II errors. Nevertheless, the sample size remains modest, which may limit the precision of effect estimates and generalizability. Fourth, functional and metabolomic validation was lacking. In addition, several potentially relevant variables were not recorded or included, including standardized histological activity indices (e.g., Geboes Score or Nancy Histology Index), dietary habits, nutritional supplementation, medication exposure beyond the 3-month exclusion window, serum albumin, and other metabolic markers. These unmeasured or unavailable factors may have introduced residual confounding. Therefore, large-scale prospective studies incorporating shotgun metagenomic sequencing and functional metabolomic validation are warranted.

## 5 Conclusions

In this cross-sectional study of UC patients, 37.2%–42.3% of the *Enterobacterales* lineage-MES association was statistically accounted for by bilirubin. These findings suggest that bilirubin metabolism may be a relevant link between gut microbiota dysbiosis and UC disease activity. Prospective studies are needed to establish the temporal and causal direction of this relationship.

## Data Availability

The datasets generated during this study are available in NCBI BioProject database, [BioProject ID: PRJNA1540835].

## References

[1] Windsor JW Coward S Hracs L Mak JWY Banerjee R Kotze PG. Trends in the ulcerative colitis to Crohn’s disease incidence ratio are distinct between epidemiologic stages of inflammatory bowel disease. Am J Gastroenterol. (2026) 121(5):1276–80. 10.14309/ajg.0000000000003868 PMC13127728 41284467

[2] Le Berre C Honap S Peyrin-Biroulet L. Ulcerative colitis. Lancet. (2023) 402(10401):571–84. 10.1016/S0140-6736(23)00966-2 37573077

[3] Gilliland A Chan JJ De Wolfe TJ Yang H Vallance BA. Pathobionts in inflammatory bowel disease: origins, underlying mechanisms, and implications for clinical care. Gastroenterology. (2024) 166(1):44–58. 10.1053/j.gastro.2023.09.019 37734419

[4] Khorsand B Asadzadeh Aghdaei H Nazemalhosseini-Mojarad E Nadalian B Nadalian B Houri H. Overrepresentation of *Enterobacteriaceae* and *Escherichia coli* is the major gut microbiome signature in Crohn’s disease and ulcerative colitis; a comprehensive metagenomic analysis of IBDMDB datasets. Front Cell Infect Microbiol. (2022) 12:1015890. 10.3389/fcimb.2022.1015890 36268225 PMC9577114

[5] Laryushina Y Samoilova-Bedych N Turgunova L Kozhakhmetov S Alina A Suieubayev M. Alterations of the gut microbiome and TMAO levels in patients with ulcerative colitis. J Clin Med. (2024) 13(19):5794. 10.3390/jcm13195794 39407853 PMC11477140

[6] Park H Yeo S Lee T Han Y Ryu CB Huh CS. Culture-based characterization of gut microbiota in inflammatory bowel disease. Front Microbiol. (2025) 16:1538620. 10.3389/fmicb.2025.1538620 40051478 PMC11884817

[7] Scaldaferri F D’Onofrio AM Chiera E Gomez-Nguyen A Ferrajoli GF Di Vincenzo F. Impact of psychopathology and gut microbiota on disease progression in ulcerative colitis: a five-year follow-up study. Microorganisms. (2025) 13(6):1208. 10.3390/microorganisms13061208 40572096 PMC12195093

[8] Kuerbannaimu K Zhao J Mukaidaisi A Wang H Zhu J Pan W. [E.faecium QH06 alleviates TNBS-induced colonic mucosal injury in rats]. Nan Fang Yi Ke Da Xue Xue Bao. (2022) 42(7):976–87. 10.12122/j.issn.1673-4254.2022.07.03 35869759 PMC9308865

[9] Hu J Tong Y Shen Z Li Y Cheng C Au R. Gegen Qinlian decoction ameliorates murine colitis by inhibiting the expansion of *Enterobacteriaceae* through activating PPAR-γ signaling. Biomed Pharmacother. (2022) 154:113571. 10.1016/j.biopha.2022.113571 36007273

[10] Zhao C Huang H Pan Q Huang W Peng W Xu H. Unconjugated bilirubin attenuates DSS-induced colitis potentially via enhancement of bilirubin reabsorption. Front Pharmacol. (2021) 12:654808. 10.3389/fphar.2021.654808 34093187 PMC8173062

[11] Yan W Du N Zhang K Yang P Guo J Xu L. Bilirubin-microbiota interaction: molecular mechanisms and therapeutic strategies in neonatal jaundice. Front Microbiol. (2026) 16:1749152. 10.3389/fmicb.2025.1749152 41608689 PMC12845330

[12] Ren Z Zhang Z Li H Fu P Wang R Wang S. Changes in serum bilirubin and total bile acids during biologic therapy in patients with ulcerative colitis: a retrospective study. J Inflamm Res. (2025) 18:9079–90. 10.2147/JIR.S524056 40672805 PMC12264352

[13] Zhang X Zeng S Cheng G He L Chen M Wang M. Clinical manifestations of neonatal hyperbilirubinemia are related to alterations in the gut microbiota. Children. (2022) 9(5):764. 10.3390/children9050764 35626941 PMC9139945

[14] Huang J Zhou B Zhu F Li Y Li Y Zhang R. Gut microbiome dysbiosis as a potential biomarker for liver metabolic disorders in in neonatal hemolytic jaundice. BMC Pediatr. (2025) 25(1):337. 10.1186/s12887-025-05692-8 40301849 PMC12039124

[15] Li J Ye S Huang X Yang G Wang Y Zeng J. Analysis of the intestinal microbiota and profiles of blood amino acids and acylcarnitines in neonates with hyperbilirubinemia. BMC Microbiol. (2024) 24(1):171. 10.1186/s12866-024-03328-y 38760685 PMC11102171

[16] Duan M Yu J Feng J He Y Xiao S Zhu D. 16S Ribosomal RNA-based gut microbiome composition analysis in infants with breast milk jaundice. Open Life Sci. (2018) 13:208–16. 10.1515/biol-2018-0025 33817085 PMC7874704

[17] Faul F Erdfelder E Buchner A Lang A-G. Statistical power analyses using G*Power 3.1: tests for correlation and regression analyses. Behav Res Methods. (2009) 41(4):1149–60. 10.3758/BRM.41.4.1149 19897823

[18] Weems CF. An alternative framework for nonexperimental cross-sectional mediation studies: associational variable analysis. Psychol Methods. (2025). [Online ahead of print]. 10.1037/met0000774 40608441

[19] Vitetta L Oldfield D Sali A. Inflammatory bowel diseases and the efficacy of probiotics as functional foods. Front Biosci (Elite Ed). (2024) 16(2):13. 10.31083/j.fbe1602013 38939911

[20] Kleessen B Kroesen AJ Buhr HJ Blaut M. Mucosal and invading bacteria in patients with inflammatory bowel disease compared with controls. Scand J Gastroenterol. (2002) 37(9):1034–41. 10.1080/003655202320378220 12374228

[21] Morgan XC Tickle TL Sokol H Gevers D Devaney KL Ward DV. Dysfunction of the intestinal microbiome in inflammatory bowel disease and treatment. Genome Biol. (2012) 13(9):R79. 10.1186/gb-2012-13-9-r79 23013615 PMC3506950

[22] He X-X Li Y-H Yan P-G Meng X-C Chen C-Y Li K-M. Relationship between clinical features and intestinal microbiota in Chinese patients with ulcerative colitis. World J Gastroenterol. (2021) 27(28):4722–37. 10.3748/wjg.v27.i28.4722 34366632 PMC8326252

[23] Rimmer P Cheesbrough J Horniblow R Tull S Iqbal A Sharma N. P84Microbial associations with disease activity at adult IBD onset. Gut. (2023) 72(Suppl 2):A96–7. 10.1136/gutjnl-2023-bsg.156

[24] Han T Zhang Y Zheng G Guo Y. From pathogenic mechanisms to therapeutic perspectives: a review of gut microbiota and intestinal mucosal immunity in inflammatory bowel disease. Front Immunol. (2025) 16:1704651. 10.3389/fimmu.2025.1704651 41306971 PMC12643992

[25] Dunleavy KA Raffals LE Camilleri M. Intestinal barrier dysfunction in inflammatory bowel disease: underpinning pathogenesis and therapeutics. Dig Dis Sci. (2023) 68(12):4306–20. 10.1007/s10620-023-08122-w 37773554 PMC10798146

[26] Abdelhalim KA Uzel A Ünal NG. The role of major virulence factors and pathogenicity of adherent-invasive *Escherichia coli* in patients with Crohn’s disease. Prz Gastroenterol. (2020) 15(4):279–88. 10.5114/pg.2020.93235 33777266 PMC7988836

[27] Shiraishi K Furukawa S Yagi S Hanayama M Mori K Ninomiya T. Association between serum bilirubin and mucosal healing among Japanese patients with ulcerative colitis: a cross-sectional study. Int J Colorectal Dis. (2021) 36(2):377–82. 10.1007/s00384-020-03774-w 33040190

[28] Zheng J-D He Y Yu H-Y Liu Y-L Ge Y-X Li X-T. Unconjugated bilirubin alleviates experimental ulcerative colitis by regulating intestinal barrier function and immune inflammation. World J Gastroenterol. (2019) 25(15):1865–78. 10.3748/wjg.v25.i15.1865 31057300 PMC6478610

[29] Baldelli V Scaldaferri F Putignani L Del Chierico F. The role of *Enterobacteriaceae* in gut microbiota dysbiosis in inflammatory bowel diseases. Microorganisms. (2021) 9(4):697. 10.3390/microorganisms9040697 33801755 PMC8066304

[30] Winter SE Winter MG Xavier MN Thiennimitr P Poon V Keestra AM. Host-derived nitrate boosts growth of *E. Coli* in the inflamed gut. Science. (2013) 339(6120):708–11. 10.1126/science.1232467 23393266 PMC4004111

[31] Li Y Huang B Ye T Wang Y Xia D Qian J. Physiological concentrations of bilirubin control inflammatory response by inhibiting NF-κB and inflammasome activation. Int Immunopharmacol. (2020) 84:106520. 10.1016/j.intimp.2020.106520 32325405

[32] Li Y Sheng H Yan Z Guan B Qiang S Qian J. Bilirubin stabilizes the mitochondrial membranes during NLRP3 inflammasome activation. Biochem Pharmacol. (2022) 203:115204. 10.1016/j.bcp.2022.115204 35944727

[33] Čepelak I Dodig S Pavić I. Bilirubin—new insights into an old molecule. Biochem Med (Zagreb). (2025) 35(2):172–85. 10.11613/BM.2025.020501 PMC12131392 40469572

[34] Milacic M Beavers D Conley P Gong C Gillespie M Griss J. The reactome pathway knowledgebase 2024. Nucleic Acids Res. (2024) 52(D1):D672–d8. 10.1093/nar/gkad1025 37941124 PMC10767911

[35] Leung JW Liu YL Leung PS Chan RC Inciardi JF Cheng AF. Expression of bacterial beta-glucuronidase in human bile: an *in vitro* study. Gastrointest Endosc. (2001) 54(3):346–50. 10.1067/mge.2001.117546 11522976

[36] Huang X Liu Y Zhou Z Pan Y Zhang Y Gao C. Clinical significance of the C-reactive protein-to-bilirubin ratio in patients with ulcerative colitis. Front Med. (2023) 10:1227998. 10.3389/fmed.2023.1227998 PMC10560853 37817808

[37] Huang X Pan Y Liu Y Zhou Z Zhang Y Gao C. Clinical utility of the neutrophil-to-bilirubin ratio in the detection of disease activity in ulcerative colitis. J Inflamm Res. (2023) 16:2549–59. 10.2147/JIR.S413644 37346801 PMC10281274

[38] Menni AE Kyriazopoulou E Karakike E Tzikos G Filidou E Kotzampassi K. Interplay of gut microbiota, biologic agents, and postoperative anastomotic leakage in inflammatory bowel disease: a narrative review. Int J Mol Sci. (2025) 26(15):7066. 10.3390/ijms26157066 40806196 PMC12345727

[39] Xu Y Xie R Weng Y Fang Y Tao S Zhang H. Role and mechanism of gut microbiota-host interactions in the pathogenesis of Crohn’s disease. Int J Colorectal Dis. (2025) 40(1):130. 10.1007/s00384-025-04917-7 40437310 PMC12119691

