# Supplementary material for: Bilirubin statistically accounts for the association between Enterobacterales lineage and Mayo endoscopic score in ulcerative colitis: a cross-sectional associational variable analysis
Source: Front Med (Lausanne). 2026 Oct 14;13:1946063. doi: 10.3389/fmed.2026.1946063 (PMC13713277; doi:10.3389/fmed.2026.1946063)
Supplement: Supplementary file 1 [file Table1.docx]

**Supplementary Table S1. Sequencing data quality control statistics for each sample**

| **Sample ID** | **Raw Reads** | **Clean Reads** | **Raw Tags** | **Clean Tags** | **Chimera** | **Effective Tags** | **Effective Ratio (%)** |
| --- | --- | --- | --- | --- | --- | --- | --- |
| U004 | 100003 | 98667 | 98502 | 98308 | 1319 | 96989 | 96.99 |
| U005 | 98718 | 97548 | 96954 | 96753 | 3253 | 93500 | 94.71 |
| U006 | 111282 | 109867 | 109212 | 108873 | 4636 | 104237 | 93.67 |
| U007 | 183238 | 181199 | 180389 | 179599 | 5629 | 173970 | 94.94 |
| U008 | 113244 | 113182 | 112191 | 111906 | 3404 | 108502 | 95.81 |
| U009 | 104568 | 103494 | 103320 | 103083 | 680 | 102403 | 97.93 |
| U013 | 87550 | 86403 | 86230 | 86073 | 706 | 85367 | 97.51 |
| U010 | 88151 | 87066 | 86944 | 86713 | 1512 | 85201 | 96.65 |
| U014 | 115740 | 114121 | 113508 | 113262 | 4278 | 108984 | 94.16 |
| U015 | 125028 | 123341 | 122506 | 121838 | 5837 | 116001 | 92.78 |
| U016 | 81424 | 80481 | 80353 | 80155 | 969 | 79186 | 97.25 |
| U018 | 100847 | 100801 | 100104 | 99653 | 6292 | 93361 | 92.58 |
| U030 | 102796 | 102737 | 101437 | 100971 | 624 | 100347 | 97.62 |
| U039 | 103873 | 103819 | 102045 | 101601 | 1803 | 99798 | 96.08 |
| U046 | 109498 | 109439 | 108479 | 108243 | 2430 | 105813 | 96.63 |
| U051 | 107714 | 107657 | 106044 | 105636 | 4080 | 101556 | 94.28 |
| U055 | 102325 | 102273 | 100514 | 100144 | 728 | 99416 | 97.16 |
| U056 | 102987 | 102942 | 101636 | 100930 | 3173 | 97757 | 94.92 |
| U058 | 106104 | 106030 | 104682 | 104231 | 5155 | 99076 | 93.38 |
| U062 | 108533 | 108437 | 107169 | 106443 | 6581 | 99862 | 92.01 |
| U065 | 121477 | 121297 | 120053 | 119544 | 18652 | 100892 | 83.05 |
| U067 | 101349 | 101295 | 99805 | 99226 | 4159 | 95067 | 93.8 |
| U068 | 108297 | 108220 | 107197 | 106921 | 4540 | 102381 | 94.54 |
| U070 | 111399 | 111344 | 109729 | 109284 | 7604 | 101680 | 91.28 |
| U072 | 111569 | 111506 | 110407 | 109619 | 6264 | 103355 | 92.64 |
| U073 | 107993 | 107926 | 106149 | 105527 | 3593 | 101934 | 94.39 |
| U074 | 101428 | 101359 | 99717 | 99312 | 4496 | 94816 | 93.48 |
| U076 | 104660 | 104595 | 103011 | 102571 | 3639 | 98932 | 94.53 |
| U079 | 106357 | 106286 | 105078 | 104714 | 3566 | 101148 | 95.1 |
| U080 | 109985 | 109911 | 108781 | 108285 | 4062 | 104223 | 94.76 |
| U088 | 122817 | 122645 | 121335 | 120729 | 17903 | 102826 | 83.72 |
| U097 | 135124 | 134931 | 133715 | 133056 | 19955 | 113101 | 83.7 |
| U102 | 123440 | 123283 | 122143 | 121601 | 13752 | 107849 | 87.37 |
| U110 | 122345 | 122133 | 120791 | 120102 | 12806 | 107296 | 87.7 |
| U111 | 120288 | 120096 | 119003 | 118557 | 21009 | 97548 | 81.1 |
| U112 | 133594 | 133427 | 132255 | 131953 | 9800 | 122153 | 91.44 |
| U113 | 128611 | 128448 | 127353 | 126744 | 15844 | 110900 | 86.23 |
| U114 | 120678 | 120488 | 119284 | 118733 | 16386 | 102347 | 84.81 |
| U115 | 132356 | 132199 | 131180 | 130668 | 19758 | 110910 | 83.8 |
| U116 | 134888 | 134746 | 133788 | 133236 | 22067 | 111169 | 82.42 |
| U117 | 135828 | 135664 | 134606 | 134273 | 15001 | 119272 | 87.81 |
| U121 | 125403 | 125225 | 123030 | 122198 | 24085 | 98113 | 78.24 |
| U122 | 129560 | 129371 | 127254 | 126665 | 19647 | 107018 | 82.6 |
| U123 | 120824 | 120626 | 118587 | 117976 | 24605 | 93371 | 77.28 |
| U124 | 122907 | 122702 | 120459 | 119936 | 23686 | 96250 | 78.31 |
| U125 | 129076 | 128848 | 126489 | 125686 | 24102 | 101584 | 78.7 |
| U126 | 123558 | 123377 | 121395 | 120721 | 18436 | 102285 | 82.78 |
| U127 | 129908 | 129688 | 127213 | 126486 | 22415 | 104071 | 80.11 |
| U128 | 123218 | 123046 | 120851 | 120246 | 27478 | 92768 | 75.29 |
| U129 | 136359 | 136180 | 133700 | 133011 | 27486 | 105525 | 77.39 |
| U130 | 126789 | 126589 | 124307 | 123633 | 20237 | 103396 | 81.55 |
| U131 | 132617 | 132418 | 130307 | 129329 | 24550 | 104779 | 79.01 |
| U132 | 127598 | 127423 | 125262 | 124385 | 23021 | 101364 | 79.44 |
| U133 | 123840 | 123656 | 121333 | 120785 | 18292 | 102493 | 82.76 |
| U134 | 129735 | 129555 | 127430 | 126663 | 23443 | 103220 | 79.56 |
| U135 | 121484 | 121290 | 119163 | 118337 | 24741 | 93596 | 77.04 |
| U136 | 70154 | 70051 | 68643 | 68279 | 15125 | 53154 | 75.77 |
| U137 | 129012 | 128830 | 126733 | 126026 | 27373 | 98653 | 76.47 |
| U138 | 125467 | 125274 | 123293 | 122509 | 27463 | 95046 | 75.75 |
| U139 | 127931 | 127717 | 125684 | 125098 | 21180 | 103918 | 81.23 |
| U140 | 131903 | 131665 | 129600 | 128873 | 29263 | 99610 | 75.52 |
| U141 | 128649 | 128405 | 126221 | 125409 | 27457 | 97952 | 76.14 |
| U142 | 133882 | 133651 | 131386 | 130713 | 22767 | 107946 | 80.63 |
| U143 | 135373 | 135162 | 132940 | 132388 | 19730 | 112658 | 83.22 |
| U144 | 130359 | 130133 | 127894 | 127122 | 17095 | 110027 | 84.4 |
| U145 | 127729 | 127516 | 125421 | 124553 | 30928 | 93625 | 73.3 |
| U146 | 126388 | 126159 | 124100 | 123579 | 26013 | 97566 | 77.2 |
| U147 | 121438 | 121234 | 119278 | 118555 | 25120 | 93435 | 76.94 |
| U148 | 133874 | 133636 | 131348 | 130527 | 27647 | 102880 | 76.85 |
| U149 | 133743 | 133554 | 131462 | 130574 | 18140 | 112434 | 84.07 |
| U150 | 128020 | 127815 | 125613 | 124822 | 28642 | 96180 | 75.13 |
| U151 | 120070 | 119875 | 118055 | 117368 | 19173 | 98195 | 81.78 |
| U152 | 122153 | 121903 | 119835 | 119118 | 24607 | 94511 | 77.37 |
| U153 | 130818 | 130595 | 128354 | 127733 | 24840 | 102893 | 78.65 |
| U154 | 136189 | 135956 | 133561 | 132830 | 30921 | 101909 | 74.83 |
| U155 | 106786 | 106579 | 104785 | 104132 | 21735 | 82397 | 77.16 |
| U156 | 125350 | 125103 | 123117 | 122793 | 13401 | 109392 | 87.27 |
| U157 | 133296 | 133016 | 130653 | 129962 | 29627 | 100335 | 75.27 |
| U158 | 130876 | 130608 | 128357 | 127776 | 25178 | 102598 | 78.39 |
| U159 | 126308 | 126056 | 124105 | 123426 | 16219 | 107207 | 84.88 |
| U160 | 136753 | 136502 | 133764 | 133074 | 14953 | 118121 | 86.38 |

**Note:** This table reports the sequencing data quality control statistics for each of the 81 samples. Raw Reads: the number of raw paired-end reads obtained from the sequencer. Clean Reads: the number of high-quality paired-end reads after quality filtering. Raw Tags: the number of tags obtained after merging paired-end reads. Clean Tags: the number of high-quality tags after quality filtering. Chimera: the number of chimeric sequences detected during OTU clustering. Effective Tags: the number of high-quality non-chimeric tags used for downstream analyses. Effective Ratio (%): the percentage of Effective Tags relative to Raw Reads, calculated as (Effective Tags / Raw Reads) × 100%. The effective read count ranged from 53,154 to 173,970 per sample, with a mean of 102,019 reads. The minimum effective read count (53,154 reads from sample U136) was used as the rarefaction depth for downstream analyses (see Supplementary Methods).

**Supplementary Table S2. Summary of tags and OTUs after taxonomic annotation for each sample**

| **Sample ID** | **Total Tags** | **Taxon Tags** | **Unclassified Tags** | **Singleton Tags** | **OTUs** |
| --- | --- | --- | --- | --- | --- |
| U004 | 96989 | 86948 | 0 | 10041 | 868 |
| U005 | 93500 | 76208 | 0 | 17292 | 1473 |
| U006 | 104237 | 78811 | 0 | 25426 | 1718 |
| U007 | 173970 | 90811 | 0 | 83159 | 1099 |
| U008 | 108502 | 93712 | 0 | 14790 | 241 |
| U009 | 102403 | 93456 | 0 | 8947 | 939 |
| U010 | 85201 | 76633 | 0 | 8568 | 771 |
| U013 | 85367 | 77602 | 0 | 7765 | 759 |
| U014 | 108984 | 77531 | 0 | 31453 | 1908 |
| U015 | 116001 | 88565 | 0 | 27436 | 1769 |
| U016 | 79186 | 71312 | 0 | 7874 | 655 |
| U018 | 93361 | 78790 | 0 | 14571 | 252 |
| U030 | 100347 | 94893 | 0 | 5454 | 267 |
| U039 | 99798 | 85347 | 0 | 14451 | 233 |
| U046 | 105813 | 94556 | 0 | 11257 | 225 |
| U051 | 101556 | 72636 | 0 | 28920 | 216 |
| U055 | 99416 | 93688 | 0 | 5728 | 259 |
| U056 | 97757 | 88524 | 0 | 9233 | 237 |
| U058 | 99076 | 82400 | 0 | 16676 | 246 |
| U062 | 99862 | 80911 | 0 | 18951 | 283 |
| U065 | 100892 | 65240 | 0 | 35652 | 369 |
| U067 | 95067 | 74691 | 0 | 20376 | 263 |
| U068 | 102381 | 89868 | 0 | 12513 | 227 |
| U070 | 101680 | 84448 | 0 | 17232 | 271 |
| U072 | 103355 | 79148 | 0 | 24207 | 360 |
| U073 | 101934 | 80925 | 0 | 21009 | 287 |
| U074 | 94816 | 81531 | 0 | 13285 | 227 |
| U076 | 98932 | 74569 | 0 | 24363 | 280 |
| U079 | 101148 | 87331 | 0 | 13817 | 240 |
| U080 | 104223 | 78145 | 0 | 26078 | 416 |
| U088 | 102826 | 65200 | 0 | 37626 | 1110 |
| U097 | 113101 | 66271 | 0 | 46830 | 521 |
| U102 | 107849 | 77064 | 4 | 30781 | 809 |
| U110 | 107296 | 71941 | 0 | 35355 | 874 |
| U111 | 97548 | 57339 | 0 | 40209 | 562 |
| U112 | 122153 | 101462 | 0 | 20691 | 358 |
| U113 | 110900 | 76783 | 0 | 34117 | 559 |
| U114 | 102347 | 71380 | 2 | 30965 | 357 |
| U115 | 110910 | 63487 | 0 | 47423 | 519 |
| U116 | 111169 | 61814 | 0 | 49355 | 473 |
| U117 | 119272 | 91576 | 0 | 27696 | 460 |
| U121 | 98113 | 76989 | 0 | 21124 | 576 |
| U122 | 107018 | 89288 | 0 | 17730 | 520 |
| U123 | 93371 | 71318 | 0 | 22053 | 554 |
| U124 | 96250 | 72746 | 0 | 23504 | 585 |
| U125 | 101584 | 79185 | 0 | 22399 | 583 |
| U126 | 102285 | 85183 | 0 | 17102 | 547 |
| U127 | 104071 | 83391 | 0 | 20680 | 556 |
| U128 | 92768 | 67665 | 0 | 25103 | 617 |
| U129 | 105525 | 79540 | 0 | 25985 | 612 |
| U130 | 103396 | 83411 | 0 | 19985 | 630 |
| U131 | 104779 | 80589 | 0 | 24190 | 632 |
| U132 | 101364 | 81608 | 0 | 19756 | 554 |
| U133 | 102493 | 83608 | 0 | 18885 | 556 |
| U134 | 103220 | 81761 | 0 | 21459 | 587 |
| U135 | 93596 | 70152 | 0 | 23444 | 533 |
| U136 | 53154 | 37298 | 0 | 15856 | 384 |
| U137 | 98653 | 75443 | 0 | 23210 | 506 |
| U138 | 95046 | 71884 | 0 | 23162 | 607 |
| U139 | 103918 | 85959 | 0 | 17959 | 509 |
| U140 | 99610 | 73855 | 0 | 25755 | 568 |
| U141 | 97952 | 72441 | 0 | 25511 | 616 |
| U142 | 107946 | 87300 | 0 | 20646 | 572 |
| U143 | 112658 | 94378 | 0 | 18280 | 552 |
| U144 | 110027 | 93960 | 0 | 16067 | 584 |
| U145 | 93625 | 65596 | 0 | 28029 | 617 |
| U146 | 97566 | 72340 | 0 | 25226 | 579 |
| U147 | 93435 | 69801 | 0 | 23634 | 710 |
| U148 | 102880 | 76755 | 0 | 26125 | 581 |
| U149 | 112434 | 97770 | 0 | 14664 | 496 |
| U150 | 96180 | 68202 | 0 | 27978 | 618 |
| U151 | 98195 | 82279 | 0 | 15916 | 555 |
| U152 | 94511 | 74070 | 0 | 20441 | 457 |
| U153 | 102893 | 82475 | 0 | 20418 | 413 |
| U154 | 101909 | 79266 | 0 | 22643 | 516 |
| U155 | 82397 | 64014 | 0 | 18383 | 430 |
| U156 | 109392 | 96964 | 0 | 12428 | 397 |
| U157 | 100335 | 75396 | 0 | 24939 | 432 |
| U158 | 102598 | 77278 | 0 | 25320 | 440 |
| U159 | 107207 | 93496 | 0 | 13711 | 402 |
| U160 | 118121 | 100801 | 0 | 17320 | 442 |
| Avg | 102019 | 79493 | 0 | 22525 | 562 |

**Note:** Total Tags: the number of effective tags (non-chimeric high-quality tags) used for OTU clustering. Taxon Tags: the number of tags that were successfully classified to a taxonomic lineage using the SILVA database. Unclassified Tags: the number of tags that could not be assigned to any known taxonomy (all zero in this dataset). Singleton Tags: the number of tags belonging to OTUs with a total abundance of one across all samples (these OTUs were filtered out prior to downstream analyses). OTUs: the number of operational taxonomic units retained after removing singletons and chimeras, clustered at 97% similarity. The last row (Avg) shows the mean values across all 81 samples.

**Supplementary Table S3. Distribution and characteristics of all detected taxa within the class *Gammaproteobacteria***

| Taxon | Taxonomic_  level | Non-zero samples | Prevalence | Mean relative abundance (%) |
| --- | --- | --- | --- | --- |
| *Gammaproteobacteria* | Class | 81 | 1 | 15.43543352 |
| *Enterobacterales* | Order | 81 | 1 | 12.80534222 |
| *Burkholderiales* | Order | 81 | 1 | 1.435796605 |
| *Pasteurellales* | Order | 79 | 0.975309 | 0.581028556 |
| *Pseudomonadales* | Order | 77 | 0.950617 | 0.280657852 |
| *Aeromonadales* | Order | 57 | 0.703704 | 0.170841654 |
| *Gemmatimonadales* | Order | 36 | 0.444444 | 0.143613284 |
| *Cellvibrionales* | Order | 10 | 0.123457 | 0.028213037 |
| *Xanthomonadales* | Order | 30 | 0.37037 | 0.019749136 |
| *Vibrionales* | Order | 41 | 0.506173 | 0.014226827 |
| *Alteromonadales* | Order | 13 | 0.160494 | 0.01301116 |
| *Steroidobacterales* | Order | 26 | 0.320988 | 0.012174049 |
| *Oceanospirillales* | Order | 19 | 0.234568 | 0.01137384 |
| *Enterobacteriaceae* | Family | 81 | 1 | 12.42892725 |
| *Pasteurellaceae* | Family | 79 | 0.975309 | 0.581028556 |
| *Morganellaceae* | Family | 78 | 0.962963 | 0.356382333 |
| *Moraxellaceae* | Family | 76 | 0.938272 | 0.184057247 |
| *Aeromonadaceae* | Family | 56 | 0.691358 | 0.167827333 |
| *Gemmatimonadaceae* | Family | 36 | 0.444444 | 0.143613284 |
| *Pseudomonadaceae* | Family | 73 | 0.901235 | 0.096600568 |
| *Nitrosomonadaceae* | Family | 37 | 0.45679 | 0.06250879 |
| *Burkholderiaceae* | Family | 64 | 0.790123 | 0.036293222 |
| *Comamonadaceae* | Family | 63 | 0.777778 | 0.033398457 |
| *Halieaceae* | Family | 10 | 0.123457 | 0.026271111 |
| *Rhodocyclaceae* | Family | 15 | 0.185185 | 0.02626337 |
| *Neisseriaceae* | Family | 49 | 0.604938 | 0.020832272 |
| *TRA3-20* | Family | 20 | 0.246914 | 0.019769753 |
| *Escherichia-Shigella* | Genus | 81 | 1 | 8.140899778 |
| *Sutterella* | Genus | 73 | 0.901235 | 0.317152333 |
| *Citrobacter* | Genus | 79 | 0.975309 | 0.223868617 |
| *Proteus* | Genus | 72 | 0.888889 | 0.189936235 |
| *Acinetobacter* | Genus | 73 | 0.901235 | 0.173258062 |
| *Aeromonas* | Genus | 56 | 0.691358 | 0.165617333 |
| *Morganella* | Genus | 58 | 0.716049 | 0.165337148 |
| *Pseudomonas* | Genus | 73 | 0.901235 | 0.096375802 |
| *Raoultella* | Genus | 67 | 0.82716 | 0.071233741 |
| *MND1* | Genus | 29 | 0.358025 | 0.050870185 |

**Note:** Prevalence was calculated as the proportion of samples in which the taxon was detected (non-zero samples / total samples, n = 81). All abundances are expressed as raw relative abundances (percentages) prior to log₁₀ transformation.

**Supplementary Table S4. Associational analysis of the *Enterobacterales* lineage with additional adjustment for inflammatory markers (hs-CRP and ESR)**

|  | *a* | *b* | *c’* | *a × b* | *c* | Proportion |
| --- | --- | --- | --- | --- | --- | --- |
| *Gammaproteobacteria* (Class level) | | | | | | |
| TBIL | −2.32 [−5.33, 0.68] | −0.06* [−0.09, −0.03] | 0.29 [−0.05, 0.63] | 0.14 [−0.05, 0.35] | 0.43 | 32.6% |
| IBIL | −2.13 [−4.39, 0.12] | −0.07* [−0.10, −0.03] | 0.29 [−0.07, 0.64] | 0.14 [−0.02, 0.36] | 0.43 | 32.6% |
| *Enterobacterales* (Order level) | | | | | | |
| TBIL | −1.55 [−4.07, 0.98] | −0.06* [−0.09, −0.04] | 0.23 [−0.05, 0.51] | 0.09 [−0.05, 0.25] | 0.32 | 28.1% |
| IBIL | −1.15 [−3.06, 0.76] | −0.07* [−0.11, −0.03] | 0.25 [−0.05, 0.54] | 0.08 [−0.06, 0.23] | 0.33 | 24.2% |
| *Enterobacteriaceae* (Family level) | | | | | | |
| TBIL | −1.64 [−4.16, 0.89] | −0.06* [−0.09, −0.04] | 0.23 [−0.05, 0.51] | 0.10 [−0.05, 0.26] | 0.33 | 30.3% |
| IBIL | −1.23 [−3.14, 0.69] | −0.07* [−0.11, −0.03] | 0.24 [−0.05, 0.54] | 0.09 [−0.05, 0.23] | 0.33 | 27.3% |
| *Escherichia-Shigella* (Genus level) | | | | | | |
| TBIL | −1.18 [−3.84, 1.47] | −0.06* [−0.09, −0.04] | 0.21 [−0.09, 0.50] | 0.07 [−0.09, 0.23] | 0.28 | 25.0% |
| IBIL | −0.94 [−2.95, 1.07] | −0.07* [−0.11, −0.04] | 0.21 [−0.09, 0.52] | 0.07 [−0.08, 0.22] | 0.28 | 25.0% |

**Note:** The coefficient *a* denotes the adjusted regression association between gut microbiota and bilirubin. The coefficient *b* denotes the adjusted regression association between bilirubin and MES after accounting for gut microbiota and the covariates. *c* denotes the total association between gut microbiota and MES The coefficient *c′* denotes the residual association between gut microbiota and MES after accounting for bilirubin and the covariates. The indirect association was calculated as *a* × *b.* The 95% CI indicates the uncertainty surrounding the estimated *a* × *b* value and was calculated using 5,000 bootstrap resamples, and the associational proportion was calculated as [(*a* × *b*) / *c*] × 100%. **P* < 0.05. Adjusted models were adjusted for age, sex, BMI, smoking history, disease duration, biologic therapy history, hs-CRP, and ESR. All estimates describe statistical covariation and should not be interpreted as causal effects. None of the indirect effects reached statistical significance, as all bootstrap 95% CIs crossed zero. TBIL, total bilirubin; IBIL, indirect bilirubin.

**Supplementary Table S5. Post‑hoc power analysis results for the MES outcome models in the associational variable analyses**

| Taxonomic level | Associative variable | R² | f² | Power | β (Type II error) |
| --- | --- | --- | --- | --- | --- |
| *Gammaproteobacteria* | TBIL | 0.428 | 0.749 | 99.9% | 0.001 |
|  | IBIL | 0.372 | 0.592 | 99.7% | 0.003 |
| *Enterobacterales* | TBIL | 0.423 | 0.735 | 99.9% | 0.001 |
|  | IBIL | 0.372 | 0.593 | 99.7% | 0.003 |
| *Enterobacteriaceae* | TBIL | 0.422 | 0.732 | 99.9% | 0.001 |
|  | IBIL | 0.371 | 0.591 | 99.7% | 0.003 |
| *Escherichia‑Shigella* | TBIL | 0.416 | 0.712 | 99.9% | 0.001 |
|  | IBIL | 0.362 | 0.568 | 99.6% | 0.004 |

**Note:** R², coefficient of determination of the MES outcome model; f², effect size calculated as f² = R² / (1 − R²); Power, statistical power computed using G*Power for multiple linear regression (F tests, fixed model, R² deviation from zero) with n = 81, nine predictors (the primary bacterial taxon, the associative variable, and seven covariates), and α = 0.05 (two‑tailed). All models exceeded the conventional 80% power threshold, indicating sufficient statistical power to detect the observed associations.

**Supplementary Figure S1. Spearman correlation heatmap of the four prespecified taxa with bilirubin and inflammatory markers.** Cell colors and values indicate Spearman correlation coefficients (ρ), with red indicating positive correlations and blue indicating negative correlations. Benjamini–Hochberg false-discovery-rate adjustment was applied across all 27 taxon–clinical variable correlation tests. *q <0.05; **q <0.01; ***q <0.001.
